# Assessment of the Anticonvulsant Potency of Ursolic Acid in Seizure Threshold Tests in Mice

**DOI:** 10.1007/s11064-018-2505-z

**Published:** 2018-03-14

**Authors:** Dorota Nieoczym, Katarzyna Socała, Piotr Wlaź

**Affiliations:** Department of Animal Physiology, Institute of Biology and Biochemistry, Faculty of Biology and Biotechnology, Maria Curie-Skłodowska University, Akademicka 19, 20-033 Lublin, Poland

**Keywords:** Ursolic acid, Seizure models, Mice

## Abstract

Ursolic acid (UA) is a plant derived compound which is also a component of the standard human diet. It possesses a wide range of pharmacological properties, i.e., antioxidant, anti-inflammatory, antimicrobial and antitumor, which have been used in folk medicine for centuries. Moreover, influence of UA on central nervous system-related processes, i.e., pain, anxiety and depression, was proved in experimental studies. UA also revealed anticonvulsant properties in animal models of epilepsy and seizures. The aim of the present study was to investigate the influence of UA on seizure thresholds in three acute seizure models in mice, i.e., the 6 Hz-induced psychomotor seizure threshold test, the maximal electroshock threshold (MEST) test and the timed intravenous pentylenetetrazole (iv PTZ) infusion test. We also examined its effect on the muscular strength (assessed in the grip strength test) and motor coordination (estimated in the chimney test) in mice. UA at doses of 50 and 100 mg/kg significantly increased the seizure thresholds in the 6 Hz and MEST tests. The studied compound did not influence the seizure thresholds in the iv PTZ test. Moreover, UA did not affect the motor coordination and muscular strength in mice. UA displays only a weak anticonvulsant potential which is dependent on the used seizure model.

## Introduction

Throughout the ages plants and their extracts were used as remedies for a wide range of disorders. Moreover, in recent times people seem to be more conscious about the usage of herbal medicines over the synthetic ones. Plant-derived compounds have served and continually serve as a source of new drugs. Important group of natural compounds with therapeutic properties are triterpenoids which include about 20,000 members and have attracted considerable interest as therapeutic compounds. Ursolic acid (UA) is a pentacyclic triterpenoid carboxylic acid present in plants in form of aglycones or glycosides. It is found in many medicinal plants (e.g., *Artemisia indica, Nepeta sibthorpii, Prunella vulgaris, Arctostaphylos uva-ursi, Vaccinium macrocarpon, Rhododendron hymenanthes*), relishes (e.g., marjoram, oregano, basil, rosemary leaves, sage, thyme, lavender, eucalyptus, black elder) as well as in wax coating some fruits (e.g., apples, pears, prunes). A wide range of pharmacological properties of UA, such as antioxidant, anti-inflammatory, antimicrobial and antitumor, has been used, even unintentionally, in plant extracts employed in folk medicine in different geographic areas [1–3]. Although UA is a regular component of the standard human diet, there is no precise information about its mean daily consumption.

In the last few decades, modern science discovered the benefits of using natural components as therapeutics and therefore plant-derived compounds are widely studied. Investigation of UA properties in experimental models showed that it might affect numerous processes in the central nervous system. The antinociceptive effect of UA was noted in the formalin test, writhing test and the model of peritoneal and/or visceral pain induced by intracolonic administration of capsaicin in mice [4]. Moreover, Bhat et al. [5] demonstrated its protective activity in chronic constriction injury-induced neuropathic pain in rats. Neuropharmacological activities of UA also include antidepressant action which was detected in the forced swim test and tail suspension test in mice and this effect was mediated by dopaminergic, serotonergic and noradrenergic systems, but not glutamatergic or opioid ones [6–8]. UA also displayed anxiolytic effect in the open field, elevated plus maze and light–dark box tests in mice [9–11]. Jeon et al. [12] demonstrated that UA prolonged sleep duration in pentobarbital-treated mice and this effect was related to increase in concentration of GABA in different brain structures. Moreover, similar results were obtained for ethanolic extract of *Prunella vulgaris*, which contains a significant amount of UA. Activation of GABAergic neurotransmitter system by this compound causes that it might be a promising candidate for insomnia therapy [12]. Experimental studies revealed that UA might be also used for the prevention/treatment of Parkinson’s disease because it showed a number of neuroprotective properties in animals treated with 1-methyl-4-phenyl-1,2,3,6-tetra hydropyridine [13]. The ability of UA to block the p38/NF-κB signaling pathways [14] and Forkhead box protein O1 as well as to activate PI3K/Akt signaling [15] might attenuate cognitive deficits in experimental models. Liang et al. [16] showed that UA significantly reduced the amyloid β-induced learning and memory deficits in mice through reduction of oxidative stress and inflammatory response, which implies that this triterpenoid may also offer a novel therapeutic strategy for the treatment of Alzheimer’s disease [16]. Anti-oxidative and anti-inflammatory properties of UA account for protection of the brain against ischemic injury. Moreover, a potent mechanism for its neuroprotective effects in mice after middle cerebral artery occlusion is upregulation of the nuclear factor-erythroid 2-related factor 2 pathway [17].

Among numerous neuropharmacological properties of UA, its anticonvulsant activity was also reported. Taviano et al. [18] noted that UA obtained from methanolic extract of *Nepeta sibthorpii*, prolonged latency to the first convulsions and decreased both number of seizures and mortality in mice treated with pentylenetetrazole (PTZ). Moreover, anticonvulsant activity was also detected for UA stearoyl glucoside isolated from *Lantana camara* in models of seizures induced by maximal electroshock and isoniazid [19]. The recent research performed with UA from methanolic extract of *Artemisia indica* supported its anticonvulsant effect in the PTZ test [10]. Although anticonvulsant properties of UA were previously investigated, there are no details referring to its influence on seizure thresholds in experimental models of seizures. Our study complements the previous knowledge about anticonvulsant properties of UA. We used three mouse models of seizures which evaluate effect of the studied compound on seizure thresholds, i.e., the 6 Hz-induced psychomotor seizure threshold test, the maximal electroshock threshold (MEST) test and the timed intravenous PTZ (iv PTZ) infusion test in mice. Moreover, we also investigated some adverse effects of UA, i.e., its influence on the motor coordination in the chimney test and on the muscular strength in the grip strength test in mice.

## Materials and Methods

### Animals

Naïve male Swiss mice weighing 23–28 g obtained from a licensed breeder (Laboratory Animals Breeding, Słaboszów, Poland) were used in the study. The animals were housed in polycarbonate cages under strictly controlled conditions (ambient temperature 21–24 °C, relative humidity 45–65%, a 12/12 light/dark cycle with the light on at 6:00 a.m., chow pellets and tap water continuously available). Mice were used in the study after at least 1 week of acclimatization. All experiments were performed at the same time of day (between 8:00 a.m. and 3:00 p.m.) to minimize circadian influences. Control and drug experiments were always done on the same day to avoid day-to-day variations in convulsive susceptibility. All procedures were conducted in accordance with the European Union Directive of 22 September 2010 (2010/63/EU) and Polish legislation acts concerning animal experimentations. The experimental procedures and protocols were approved by the Local Ethics Committee of Lublin (26/2016).

### Drugs

UA (Toronto Research Chemicals Inc., Toronto, ON, Canada) was suspended in 1% aqueous solution of Tween 80 (POCH, Gliwice, Poland) and injected intraperitoneally (ip) in a volume of 0.1 ml per 10 g body weight 120 min prior to the tests. The pretreatment time was established during the initial experiment which examined the time-course effect of UA in the 6 Hz seizure threshold test in mice. In order to verify the reliability of the seizure tests, we additionally evaluated the influence of sodium valproate (VPA, at a dose of 150 mg/kg in the iv PTZ and MEST tests and at a dose of 50 mg/kg in the 6 Hz test) on seizure thresholds (as positive control). The used doses of VPA were selected according to our previous studies [20]. VPA (as sodium salt; Sigma-Aldrich Co., St. Louis, MO, USA) was dissolved in saline and administered ip 15 min before the tests. All drug solutions/suspensions were prepared freshly. Control animals (negative control) received 1% aqueous solution of Tween 80 at appropriate volume and time.

### The 6 Hz Electroshock-Induced (Psychomotor) Seizures

Psychomotor seizures (6-Hz seizures) were induced via corneal stimulation (0.2 ms square pulses at 6 Hz for 3 s) using a Grass S48 stimulator coupled with a constant current unit CCU1 (both from Grass Technologies, West Warwick, RI, USA). An ocular anesthetic, 1% solution of tetracaine hydrochloride, was placed on the animals’ corneas before the stimulation. The electrodes were soaked in 0.9% saline immediately before testing to ensure a good electrical contact. Mice were restrained manually during stimulation and placed in a polycarbonate box (35 × 20 × 14 cm) for observation immediately after the stimulation. Psychomotor seizures were characterized by stun, which was often followed by rearing, forelimb clonus, and twitching of vibrissae, Straub-tail, which lasted at least 10 s from the stimulation [21]. The animal was considered to be protected if it resumed its normal exploratory behavior within 10 s from the stimulation.

To determine the time-course of anticonvulsant effect of UA in the 6 Hz test in mice, the compound was injected ip at a fixed dose of 50 mg/kg and tested at 15, 30, 60, 120 and 240 min post-injection time points. In this experiment we used a relatively high dose of the studied triterpenoid to detect its potential anticonvulsant effect. To evaluate the dose–response relationship for UA in the 6 Hz test, animals were treated with doses ranging from 10 to 100 mg/kg of the studied compound and tested 120 min after the injection (at the time point in which UA showed statistically significant anticonvulsant effect). The negative control group was treated with 1% aqueous solution of Tween 80 120 min before the test while the positive control group received VPA at a dose of 50 mg/kg 15 min before the 6 Hz test. Experimental groups in the 6 Hz test consisted of 18–20 animals.

The mice were subjected to stimuli with different current intensities according to the “up-and-down” method [22]. Each mouse was stimulated only once at any given current intensity (8–23 mA) and convulsant activity was judged as described above. If the mice responded with seizures, the next mouse was stimulated with a current of an intensity 0.06-log step lower than the previous one. If the mouse did not exhibit seizures, the next one was stimulated with a current of an intensity 0.06-log step higher than the previous one. The median current strength (CS_50_ in mA; the current strength of 6 Hz stimulation which induce psychomotor seizures in 50% of the tested animals) with the standard error of the mean (SEM) was calculated as described elsewhere [22].

CS_50_ values (with their SEM) were analyzed with one-way analysis of variance (ANOVA) followed by the Tukey’s post hoc test for multiple comparisons.

### The Maximal Electroshock Seizure Threshold (MEST) Test

The seizures were induced by applying a sine-wave alternating current (maximal output voltage 500 V, 50 Hz for 0.2 s) via transcorneal electrodes. A constant current stimulator (Rodent Shocker, Type 221, Hugo Sachs Elektronik, Freiburg, Germany) was used. Ocular anesthetic (1% solution of tetracaine hydrochloride) was applied to animals’ eyes before stimulation and 0.9% saline was used to wet electrodes before testing to provide good electrical contact. Animals were manually restrained during the stimulation and immediately after the stimulation, they were placed in a polycarbonate box (35 cm × 20 cm × 14 cm) and observed for the presence or absence of the maximal (tonic) hindlimb extension.

The threshold for maximal seizures was determined according to the above-mentioned method described by Kimball et al. [22]. Each mouse was stimulated only once at any given current intensity. The data obtained in groups of 18–20 mice were used to determine the threshold current intensity which produces the hindlimb extension in 50% of the animal tested (CS_50_ in mA). Animals in the negative control group were treated with 1% aqueous solution of Tween 80 (120 min before the test) and the positive control group was injected with VPA at a dose of 150 mg/kg (15 min before the test). UA was administered at doses ranging from 10 to 100 mg/kg.

One-way ANOVA followed by the Tukey’s post hoc test for multiple comparisons was used to compare CS_50_ values.

### The Timed iv PTZ Infusion Test in Mice

At the appropriate time after UA (10–100 mg/kg), VPA (150 mg/kg, positive control) and 1% aqueous solution of Tween 80 (negative control) administration, mice were placed in the restrainer and a needle (27G, ¾ in., Sterican®, B. Braun Melsungen AG, Melsungen, Germany) was inserted in the lateral tail vein. The needle was connected by polyethylene tube (PE20RW, Plastics One Inc. Roanoke, VA, USA) with a plastic syringe that was placed in the syringe pump (Harvard Apparatus, Holliston, MA, USA). The syringe contained 1% solution of PTZ in saline, which was administered into the vein of unrestrained animal at a constant rate of 0.2 ml/min. The time intervals from the start of infusion of PTZ solution to the appearance of three separate endpoints, i.e., the first myoclonic twitch, generalized clonus with loss of righting reflex and forelimb tonus, were recorded. The threshold, in mg of PTZ per kg of body weight, for each endpoint was calculated according to the following formula:

$${\text{PTZ }}\left( {{\text{mg/kg}}} \right){\text{ }}=\frac{{{\text{infusion}}\,{\text{duration}}{\kern 1pt} \,({\text{s}})\;\, \times \;\,{\text{infusion}}\,{\text{rate}}\,({\text{ml/s}})\,\; \times \;\,{\text{PTZ}}\,{\text{concentration}}\,({\text{mg/ml}})}}{{{\text{weight}}\,{\text{(kg}})}}$$

The data obtained in groups of 11–15 mice were presented as mean ± SEM threshold dose of PTZ in mg/kg for the first myoclonic twitch, generalized clonus with loss of righting reflex and forelimb tonus, and analyzed with one-way analysis of variance (ANOVA) followed by the Tukey’s multiple comparison test.

### The Chimney Test

Chimney test [23] was used to detect the motor deficits in mice induced by UA. In this test, the inability of animals to climb back-ward up through a Plexiglas tube (3 cm, inner diameter × 30 cm, length) within 60 s is an indicator of motor impairment. The results were presented as the percent of impaired mice in a group. Each group consisted of 12 mice and the Fisher’s exact probability test was used for statistical analysis of the data.

### The Grip-Strength Test

Influence of UA on muscular strength in mice were determined in the grip-strength test [24]. The apparatus for this test consisted of a steel wire grid (8 × 8 cm) connected to an isometric force transducer. Mice were lifted by their tail so that they grasp the grid with their forepaws. The mice were then gently pulled backward until they released the grid and the maximal force exerted by the mouse before losing the grip was measured. The mean of three consecutive measurements for each animal was calculated and normalized to body weight (mN/g). Each experimental group consisted of 12 animals.

One-way ANOVA was used for statistical analysis of the data.

## Results

### Time-Course and Dose–Response Relationship for UA in the 6 Hz Seizure Test in Mice

**Fig. 1:**
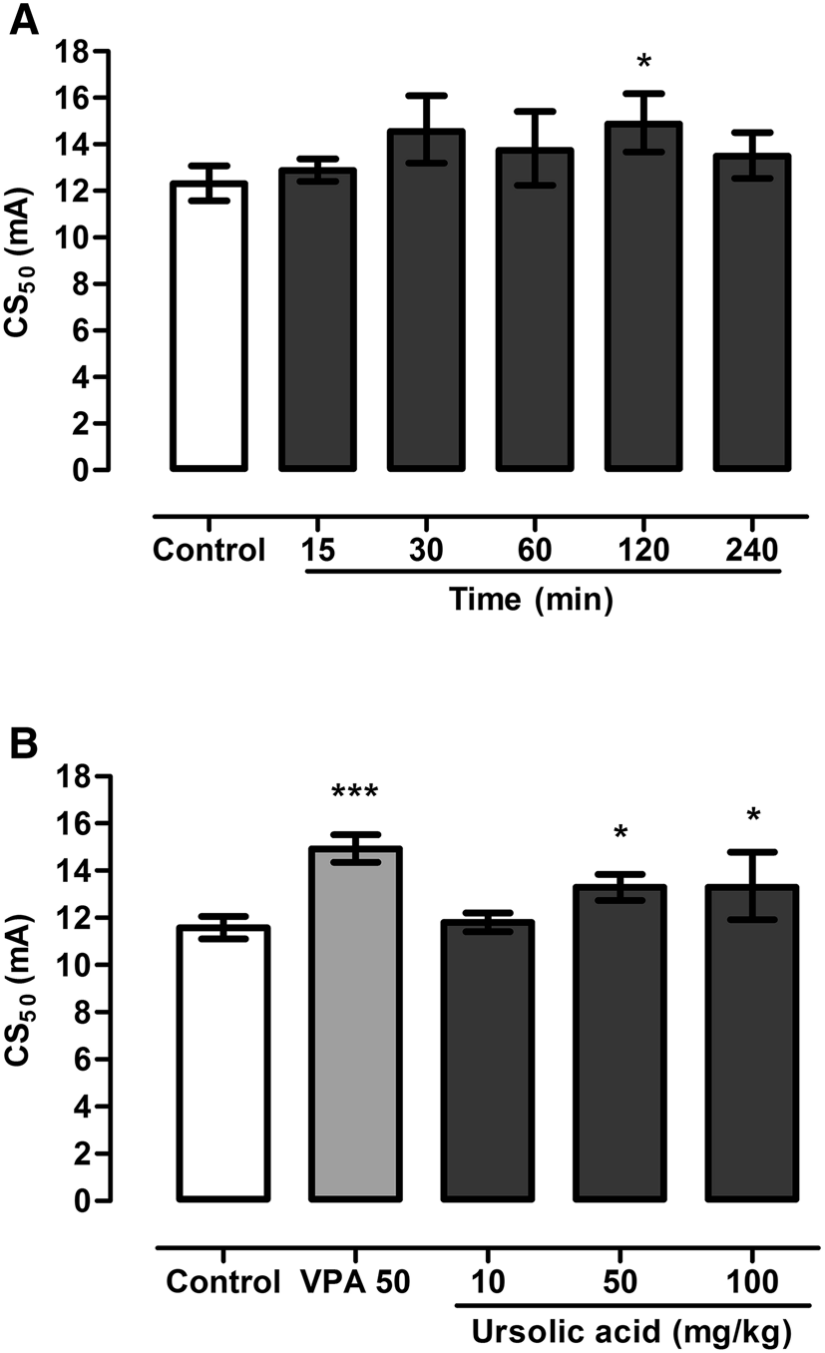
Time-course (**a**) and dose–response relationship (**b**) for UA in the 6 Hz seizure test in mice. Results are presented as median current strengths (CS_50_ in mA with their 95% confidence limits) required to produce psychomotor seizures in 50% of animal tested. UA was administered ip at a dose of 50 mg/kg at various pretreatment times (15, 30, 60, 120 and 240 min prior to the test) to evaluate time-course of its anticonvulsant effect. Dose response relationship for UA was determined for doses ranging from 10 to 100 mg/kg which were administered 120 min before the test. VPA (at a dose of 50 mg/kg, positive control) was administered ip 15 min before the test and tested to verify reliability of the method. One-way ANOVA followed by the Tukey’s post-hoc multiple comparison test was used to analyze the data.*p < 0.05 and ***p < 0.001 versus negative control group

UA administered ip at a dose of 50 mg/kg increased seizure threshold in the 6 Hz seizure test in mice. Noticeable changes were visible 30, 60 and 120 min after its administration. UA injected 120 min before the seizure test enhanced seizure threshold from 12.30 (11.58–13.07) mA in the control group to 14.88 (13.68–16.17) mA and it was the only statistically significant change (Fig. 1a, one-way ANOVA: F(5,49) = 2,965; P = 0.0204).

Based on the results from the above experiment, UA was administered ip 120 min before the 6 Hz test at doses ranging from 10 to 100 mg/kg. Statistically significant changes in seizure thresholds were noted in groups which were treated with doses of 50 and 100 mg/kg (Fig. 1b, one-way ANOVA: F(4,42) = 13.19; P < 0.0001). Seizure threshold in the control group was 11.57 (11.01–12.06) mA while in group which was injected with UA at a dose of 50 mg/kg it was raised by ~15% (p < 0.05 vs. negative control group). Statistically significant increase in seizure threshold compared to the control group was also noted in group which received UA at a dose of 100 mg/kg (p < 0.05 vs. negative control group), however this change was not statistically greater than in group treated with UA at a dose of 50 mg/kg. VPA at a dose of 50 mg/kg (positive control) increased seizure threshold to 14.93 (14.35–15.53) mA (p < 0.001 vs. negative control group).

### Effect of UA in the MEST Test in Mice

**Fig. 2:**
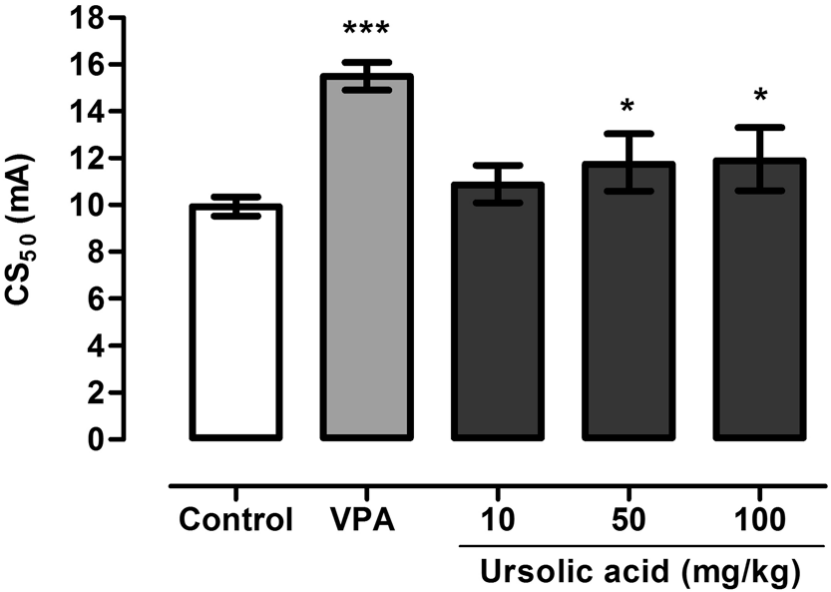
Effect of UA on seizure threshold in the MEST test in mice. UA was administered 120 min prior to the test at doses ranging from 10 to 100 mg/kg. VPA (positive control) was injected 15 min before the test at a dose of 150 mg/kg and tested to verify reliability of the method. Results are presented as median current strengths (CS_50_ in mA with their 95% confidence limits) required to produce tonic hindlimb extension in 50% of animal tested. One-way ANOVA followed by the Tukey’s post-hoc multiple comparison test was used to analyze the data. *p < 0.05 and ***p < 0.001 versus negative control group

One-way ANOVA showed that UA significantly increased seizure thresholds in the MEST test in mice (Fig. 2; F(4,40) = 18,17, p < 0.0001). In control group seizure threshold was 9.92 (10.34–9.52) mA while in groups treated with the studied compound at doses of 50 and 100 mg/kg it was increased by ~18 and ~ 20%, respectively. In group of animals treated with VPA at a dose of 150 mg/kg (positive control) CS_50_ value was 15.49 (16.10–14.90) mA (p < 0.0001 vs. negative control group).

### Effect of UA in the Timed iv PTZ Infusion Test in Mice

**Fig. 3:**
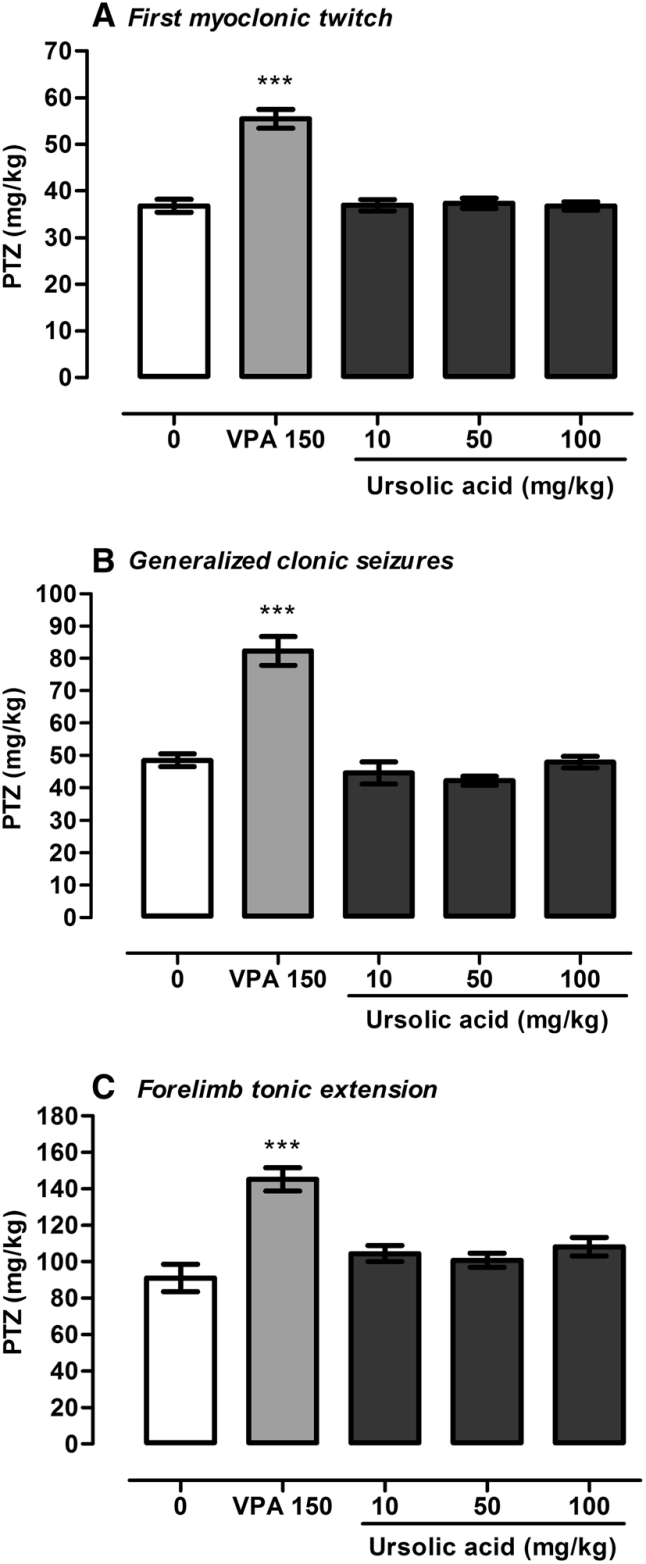
Effect of UA on the thresholds for the first myoclonic twitch (**a**), generalized clonus with loss of righting reflex (**b**) and forelimb tonic extension (**c**) in the iv PTZ test in mice. UA was administered 120 min prior to the test at doses ranging from 10 to 100 mg/kg. VPA (positive control) was injected 15 min before the test at a dose of 150 mg/kg and tested to verify reliability of the method. Data are presented as means ± SEM. One-way ANOVA followed by the Tukey’s post-hoc multiple comparison test was used to analyze the data. ***p < 0.001 versus negative control group

UA (10–100 mg/kg) did not produce any statistically significant changes in three different seizure thresholds in the iv PTZ test in mice, i.e., in the thresholds for the first myoclonic twitches (one-way ANOVA: F(4,58) = 37.08, P < 0.0001), the generalized clonic seizures (one-way ANOVA: F(4,60) = 32.59, P < 0.0001) and the forelimb tonic extension (one-way ANOVA: F(4,59) = 14.07, p < 0.0001). Statistically significant changes in seizure thresholds were observed only in a group treated with VPA at a dose of 150 mg/kg (positive control, p < 0.001). The results are shown in Fig. 3.

### Effect of UA in the Chimney and Grip-Strength Tests in Mice

One-way ANOVA did not show any statistically significant changes in the muscular strength in mice treated with VPA at a dose of 150 mg/kg as well as in mice treated with UA at doses ranging from 10 to 100 mg/kg (F(4,45) = 0.9338, p = 0.453). Moreover, neither VPA nor UA induced impairment of the motor coordination in mice (p > 0.05) which was assessed in the chimney test. Results from the grip-strength and the chimney tests are presented in Table 1.

**Table 1:** Effects of UA in the grip strength and chimney tests in mice

Treatment (mg/kg)	Neuromuscular strength (mN/g)	Impairment of motor performance (%)
Vehicle (control)	30.68 ± 1.02	16.6
VPA (150)	28.72 ± 2.11	8.3
UA (10)	30.65 ± 1.43	0
UA (50)	29.25 ± 1.25	0
UA (100)	28.74 ± 0.88	0

Results are presented as mean (± SEM) grip strengths in millinewtons per gram of mouse body weight (mN/g) from the grip-strength test, assessing the neuromuscular strength in mice, and as percentage of animals showing the motor coordination impairment in the chimney test in mice. Each experimental group consisted of 12 animals. Statistical analysis of data from the grip-strength test was performed with one-way ANOVA (F(4,55) = 0.494; p = 0.74) and the Fisher’s exact probability test (p > 0.05) was used to analyze the results from the chimney test

## Discussion

In the present study, we evaluated the effect of UA on seizure thresholds in three acute seizure models in mice. Two of these models, i.e., the 6 Hz psychomotor seizure and MEST tests, involved electrical stimulation to induce convulsions in mice. We also used a test in which seizures were induced by chemoconvulsant—the timed iv PTZ infusion test. The above mentioned tests, especially the MEST and PTZ tests, are widely used in experimental research on compounds with anticonvulsant properties [25]. The 6 Hz model was underestimated for many years due to inefficacy of phenytoin in this test and it attracted attention only after discovery of levetiracetam. The 6 Hz test is currently included in the Anticonvulsant Drug Development (ADD) Program of the National Institute of Neurological Disorders and Stroke (NINDS) of the National Institutes of Health (NIH; Bethesda, MD) to screen efficacy and early detection of anticonvulsant activity [26]. The used models allow to evaluate the influence of UA on thresholds for different types of seizures because the MEST test is considered as a model of generalized tonic-clonic seizures, the PTZ-induced seizures are associated with absence seizures while the 6 Hz test is a screening test for partial seizures [27, 28]. Moreover, the test of the maximal electroshock allows to identify compounds which prevent seizure spread in the central nervous system [29].

Natural compounds have been widely studied in hopes of finding effective drugs for many diseases, including disorders of the nervous system. They seem to be a good source of therapeutics due to their low toxicity. Among natural compounds which could affect functions of the central nervous system are pentacyclic triterpenoids, for example asiatic, oleanolic, betulinic, boswellic and ursolic acid [30]. Neuroprotective properties of these compounds were investigated in different models of seizures and epilepsy and protective properties were noted, among others, for UA [10, 19]. Although effects of UA on the central nervous system have been studied extensively, there are no studies demonstrating its time-course effect. We evaluated the time-course effect of UA in the 6 Hz test in mice and for the first time demonstrated that its highest pharmacological activity is visible 120 min after ip administration. In most studies, UA was administered 30 min [11, 12] or 60 min [8, 18] before tests.

Anticonvulsant activity of UA was evaluated for the first time by Taviano et al. [18] in model of seizures induced by ip injection of PTZ in mice. UA acquired from *Nepeta sibthorpii* and administered intragastrically at a dose of 2.3 mg/kg significantly prolonged latency to the first seizure as well as decreased the number of seizures and animal mortality [18]. Recently, Khan et al. [10] have also investigated effects of UA in the ip PTZ test in mice. They used UA extracted from *Artemisia indica* and administered it ip at considerably higher doses (1–100 mg/kg) than Taviano et al. [18]. In this case, UA at doses ranging from 10 to 100 mg/kg significantly increased latency to clonic-tonic seizures, decreased their duration and mortality. In our study, UA was investigated in the iv PTZ which is considered as a very sensitive model which allows to examine effect of the studied compounds on three different kinds of seizures [29]. Infusion of PTZ at a constant rate into the tail vain elicits reproducible seizure activity, i.e., the first myoclonic twitch, generalized clonus with loss of righting reflex and forelimb tonus which is often followed by animal death. This test provides detailed information about seizure susceptibility and respective kinds of seizures in individual animals [29]. Moreover, it might be used not only to detect anticonvulsant effect but also proconvulsant activity. Comparison of two routes of PTZ administration, i.e., iv and subcutaneous (sc), revealed differences in delivery of PTZ to brain which result in different expression of seizures in these two models. In the sc PTZ test the brain uptake of PTZ is gradual, which is connected to its gradual absorption from site of injection. In iv PTZ administration, phase of absorption is omitted, PTZ concentration in brain increases more uniformly and rapidly. Moreover, a comparative study of PTZ induced seizures in rats, revealed differences in the brain regions which are activated during seizure initiation in the iv and ip PTZ tests. The studies using the functional magnetic resonance imaging demonstrated that iv PTZ administration produces single dominant cluster which involves the majority of the brain. In case of ip administration of PTZ multiple and relatively small clusters were noted [31]. There were also observed differences in activity of antiepileptic drugs in the iv and sc PTZ tests in mice because levetiracetam and pregabalin were effective in the iv PTZ test but did not have significant anticonvulsant effect in the sc PTZ test in mice [29]. Taking into account the above differences, it could be assumed that the mechanism of induction of convulsions in the PTZ tests shows differences, which might also explain dissimilarity of results presented in our study and the previous studies [10, 18].

The maximal electroshock-induced seizures is one of the most commonly used paradigm to identify drugs with potential activity against generalized tonic-clonic seizures in humans [32]. The endpoint in this test is the tonic hindlimb extension which is induced by transcorneal or transauricular supramaximal electrical stimulation in normal nonconvulsive mice. It is thought that tonic hindlimb extension is abolished mainly by drugs blocking voltage dependent sodium channels although majority of standard and newly developed antiepileptic drugs also exhibit protective action in this test. The conventional maximal electroshock test allows identification of compounds which are potent enough to rise seizure threshold above 50 mA while the MEST test gives the opportunity to evaluate also components with weaker anticonvulsant properties and therefore the MEST procedure is also often used in experimental studies [33]. Kazmi et al. [19] noted that UA stearoyl glucoside administered ip at doses of 25 and 50 mg/kg significantly reduced duration of tonus hindlimb extension and mortality in the maximal electroshock test in rats. To complement that study we did not use the maximal electroshock test in its standard form with fixed supramaximal current delivery, but we used the threshold test to assess influence of UA on seizure threshold, i.e., current strength which induce the tonic hindlimb extension in 50% of the tested animals. Our results revealed that UA (doses of 50 and 100 mg/kg) raised seizure threshold in a statistically significant manner in this test although noted increases were modest.

The third model that we used was model of the psychomotor seizures induced by 6 Hz stimulation in mice. This test serves as a model of human partial complex seizures and was also considered as a potent model of pharmacoresistant limbic seizures in humans [21]. However, the validity of this theory has been undermined recently because some clinically established anticonvulsant drugs, which are efficacious in protecting against 6 Hz seizures induced by 44 mA stimulation, are not potent in patients with refractory seizures [32]. Our study demonstrated for the first time the anticonvulsant properties of UA (doses of 50 and 100 mg/kg) in the 6 Hz test in mice.

The present study extends our knowledge about anticonvulsant action of UA in animal models of seizures. Using three different acute seizure induction methods that model varying types of seizures allows us to predict some mechanism of anticonvulsant action of UA. Although there are some reports which showed that UA enhance GABAergic transmission [10, 12] we did not note its anticonvulsant action in the PTZ test. Instead, anticonvulsant effect of UA was observed in the MEST and 6 Hz tests which might suggest that enhancement of GABAergic neurotransmission is not one and only mechanism of its anticonvulsant action. It is probable that UA blocks voltage sodium channels and/or possesses multiple anticonvulsant mechanisms. Although UA does not exhibit significant anticonvulsant activity which might be useful in therapy of patients with epilepsy, its mechanisms of action should be further investigated for better understanding its effects on other functions of central nervous system. Moreover, our results might be also significant suggestion in looking for new compounds with relevant anticonvulsant activity and molecular structure similar to UA.

The chimney and grip-strength tests did not revealed any adverse effects of UA. Although results of experimental studies with animal models could not be extrapolated directly to human, we could hypothesize that this compound should not impair motor coordination and muscular strength in humans. Moreover, Bang et al. [34] showed that UA supplementation (at a dose of 450 mg/day) during resistance training improved muscle strength in men.

In conclusion, our results confirmed the previous reports on anticonvulsant potential of UA. However, effects in different seizure models are very varied and dependent on the used doses, time of administration and seizure model. The mechanism of anticonvulsant action of UA is unclear and seems to be complex and therefore should be further investigated.

## Abbreviations

CS_50_: Current strength required to induce seizure response in 50% of mice
ip: Intraperitoneally
iv: Intravenously
MEST: Maximal electroshock seizure threshold
PTZ: Pentylenetetrazole
sc: Subcutaneously
SEM: Standard error of the mean
UA: Ursolic acid
